# Structure and Self-Assembly of the Calcium Binding Matrix Protein of Human Metapneumovirus

**DOI:** 10.1016/j.str.2013.10.013

**Published:** 2014-01-07

**Authors:** Cedric Leyrat, Max Renner, Karl Harlos, Juha T. Huiskonen, Jonathan M. Grimes

**Affiliations:** 1 Division of Structural Biology, The Wellcome Trust Centre for Human Genetics, University of Oxford, Roosevelt Drive, Oxford OX3 7BN, UK; 2 Diamond Light Source Limited, Harwell Science and Innovation Campus, Didcot, Oxfordshire, OX11 0DE, UK

## Abstract

The matrix protein (M) of paramyxoviruses plays a key role in determining virion morphology by directing viral assembly and budding. Here, we report the crystal structure of the human metapneumovirus M at 2.8 Å resolution in its native dimeric state. The structure reveals the presence of a high-affinity Ca^2+^ binding site. Molecular dynamics simulations (MDS) predict a secondary lower-affinity site that correlates well with data from fluorescence-based thermal shift assays. By combining small-angle X-ray scattering with MDS and ensemble analysis, we captured the structure and dynamics of M in solution. Our analysis reveals a large positively charged patch on the protein surface that is involved in membrane interaction. Structural analysis of DOPC-induced polymerization of M into helical filaments using electron microscopy leads to a model of M self-assembly. The conservation of the Ca^2+^ binding sites suggests a role for calcium in the replication and morphogenesis of pneumoviruses.

## Highlights

• M is a calcium binding protein
• Calcium stabilizes the structure of M
• M forms an obligate dimer in solution
• M self-assembles in the presence of lipids
• The Paramyxoviruses and the Filoviruses have a common ancestor

## Teaser

The matrix protein (M) of paramyxoviruses plays a key role in determining virion morphology by directing viral assembly and budding. Leyrat et al. show that M forms an obligate dimer stabilized by calcium ion binding and suggest a role for Ca^2+^ in the replication and morphogenesis of some paramyxoviruses.

## Introduction

Human metapneumovirus (HMPV) is a leading cause of acute respiratory diseases in children, the elderly, and immune-compromised patients worldwide (Boivin et al., 2002, van den Hoogen, 2007, van den Hoogen et al., 2003, Williams et al., 2004, Xepapadaki et al., 2004). Together with respiratory syncytial virus (RSV), HMPV is grouped into the *Pneumovirinae* subfamily of the *Paramyxoviridae* (van den Hoogen et al., 2002). HMPV is an enveloped virus with an ∼13-kb, single-stranded (−) RNA genome that encodes nine proteins in the order 3′-N-P-M-F-M2(−1)/(−2)-SH-G-L-5′. HMPV proteins show detectable levels of sequence identity to RSV, but the order of the genes is different and HMPV lacks the NS1 and NS2 genes present in RSV. For all paramyxoviruses, the nucleoprotein (N) encapsidates viral RNA, leading to an N-RNA complex, and forms with the RNA-dependent RNA polymerase (L) and the phosphoprotein (P) the viral replication complex. The matrix protein (M) is a major component of the virus, and is thought to form an ordered layer beneath the viral membrane (Battisti et al., 2012, Liljeroos et al., 2013). The M2 gene is specific to the *Pneumovirinae* subfamily and possesses two overlapping open reading frames encoding two proteins, the antitermination/transcription elongation factor M2-1, which is required for viral transcription (Fearns and Collins, 1999), and the RNA synthesis regulatory factor M2-2 (Buchholz et al., 2005).

HMPV virions bud from the cell surface and form pleiomorphic or filamentous particles (Peret et al., 2002). The viral membrane contains the three viral transmembrane glycoproteins (G, F, and SH), along with the matrix protein (M), which associates with the membrane’s inner surface. M plays a critical role in assembly and budding through interactions with multiple viral and cellular components such as nucleoprotein-RNA oligomers (N-RNA; Ghildyal et al., 2002), lipid membranes (McPhee et al., 2011), and cytoplasmic tails of the viral glycoproteins (Henderson et al., 2002). In addition, viral matrix proteins are known to possess immunomodulatory properties through interactions with nucleic acids and host cell proteins, nucleocytoplasmic trafficking, and inhibition of host cell transcription (reviewed in Ghildyal et al., 2006).

Among the order *Mononegavirales*, X-ray crystallographic structures of M proteins have been solved for RSV (*Pneumovirus-Paramyxoviridae*; Money et al., 2009), Newcastle disease virus (NDV; A*vulavirus*-*Paramyxoviridae*; Battisti et al., 2012), and Ebola virus (EBOV; Dessen et al., 2000), which belongs to the distantly related *Filoviridae* family but nevertheless possesses an M protein that is structurally related to RSV M (Money et al., 2009). Additionally, the crystal structure of Borna disease virus (BDV) M (Neumann et al., 2009) from *Bornaviridae* and the structure of several Ms from members of the *Rhabdoviridae* family have been solved (Gaudier et al., 2002, Graham et al., 2008). Contrary to M proteins of *Paramyxoviridae* and *Filoviridae*, these proteins possess a single-domain M protein. However, BDV M has been shown to be homologous to the N-terminal domain (NTD) of EBOV VP40, suggesting that *Paramyxoviridae* and *Filoviridae* M proteins evolved by gene duplication Neumann et al. (2009). Interestingly, while EBOV and RSV M have been crystallized as monomers, NDV and BDV Ms form dimers and tetramers, respectively, with a similar quaternary diamond shape.

In this study, we solved the X-ray crystallographic structure of the M protein from HMPV at 2.8 Å resolution. Furthermore, we analyzed the solution structure of intact M using small-angle X-ray scattering (SAXS) combined with classical, microsecond-long, explicit solvent molecular dynamics simulations (MDSs) and the ensemble optimization method (EOM). We show that HMPV M is a dimer, both in the crystal and in solution, and Ca^2+^ stabilizes the structure. Similarly to RSV M, HMPV M assembles into helical filaments in the presence of lipids. An electron microscopy reconstruction of the ultrastructure of an M filament allows us to propose a model of M assembly in the virion. Finally, the similarity with M proteins from other paramyxoviruses and filoviruses enables evolutionary relationships between these different viruses to be discerned.

## Results

### Crystal Structure of HMPV M

HMPV M was recombinantly expressed in *E. coli*, with an N-terminal small ubiquitin-like modifier (SUMO) tag followed by a 3C protease cleavage site. The tag was essential for soluble expression and maintaining the protein in solution. The stringent buffer conditions required for untagged M solubility (see Experimental Procedures) resulted in slow and incomplete cleavage of the SUMO tag, which further led to additional degradation of untagged M as observed by SDS-PAGE (data not shown), presumably in loop regions. HMPV M was crystallized from a mixture of intact and proteolyzed untagged M purified on gel filtration after prolonged incubation with 3C protease at 4°C, and this led to some irreproducibility in crystallization. HMPV M was solved at 2.8 Å resolution by molecular replacement using the structure of RSV M (Protein Data Bank ID [PDB ID] 2VQP; sequence identity, 38%). Data collection and refinement statistics are given in Table 1 (R_work_ = 0.19; R_free_ = 0.23).

**Table 1:** Crystallographic Statistics

Data Collection	
Beamline	Diamond I03
Wavelength (Å)	0.97949
Space group	P3_1_
Unit cell constants (Å)	a = b = 62.0, c = 275.4
Resolution limits (Å)^a^	53.7–2.8 (3.0–2.8)
Number of measured reflections	269,370
Number of unique reflections	27,947
Completeness of data (%)	99.5 (99.1)
R_merge_ (%)^b^	19.4 (181.5)
R_pim_ (%)	6.6 (60)
Multiplicity	9.6 (9.9)
I/σ	9.5 (1.7)

**Refinement**	

R_xcpt_ (%)^c^	18.5
R_free_ (%)^d^	22.9
Number of atoms (protein/water/other)	6935, 174, 5
Ramachandran favored/outliers (%)	94.1/1.8
Rmsd bond length	0.012
Rmsd bond angle	1.5
Average *B* factors (protein/water/other) (Å^2^)	88, 70, 82

R_pim_, precision-indicating merging R factor; Rmsd, root mean square deviation from ideal geometry.

a The values for the highest resolution shell are given in parentheses.

b R_merge_ = Σ_hkl_ Σ_i_|I(hkl;i) − <I(hkl)>|/Σ_hkl_ Σ_i_I(hkl;i), where I(hkl;i) is the intensity of an individual measurement of a reflection and <I(hkl)> is the average intensity of that reflection.

c R_xpct_ = Σ_hkl_||F_obs_| − |F_xpct_||/Σ_hkl_ |F_obs_|, where |F_obs_| and |F_xpct_| are the observed structure factor amplitude and the expectation of the model structure factor amplitude, respectively.

d R_free_ = R_xpct_ of the test set (1%–5% of the data removed prior to refinement).

**Figure 1:**
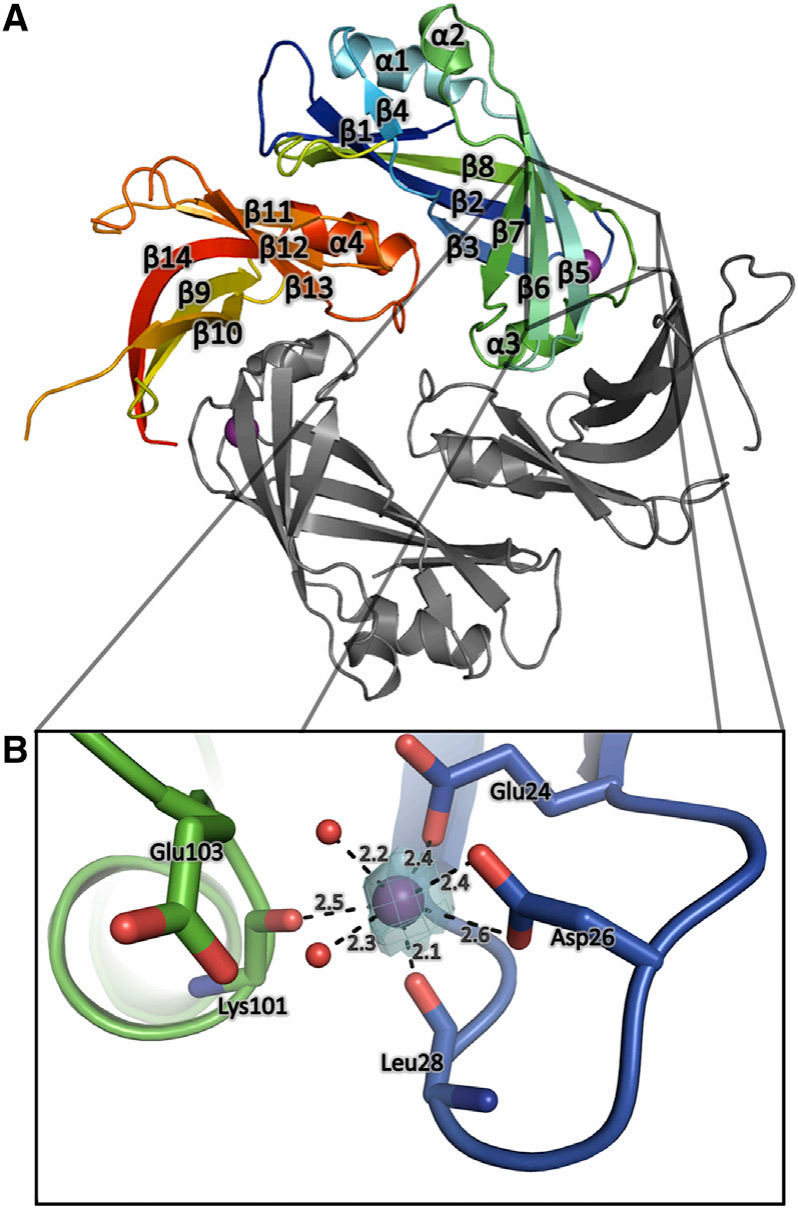
Crystal Structure of HMPV M (A) Structure of the M dimer. One of the monomers is colored from blue (N terminus) to red (C terminus) with secondary structure elements labeled, whereas the other one is in gray. (B) Close-up of the Ca^2+^ binding site. The Ca^2+^ ion is represented as a purple sphere in the electron density from an omit map (contour level = 4σ) calculated using PHASER and omitting the Ca^2+^ ion. Coordinating water molecules are displayed as nonbonded spheres. See also Figure S1.

The P3_1_ crystallographic asymmetric unit contains flattened, diamond-shaped M dimers. The M dimer is stabilized by a large network of conserved hydrophobic interactions with a buried interface area of 1,421 Å^2^ per monomer (Figure 1A; see also Figure S1 available online). Each M subunit is composed of two similarly folded domains (NTD and C-terminal domain [CTD]), which are joined by a 14-residue linker (residues 123–137) for which no density is visible. The dimeric interface involves contacts between the NTD and CTD of the related monomers. The NTD comprises residues 1–123 and the CTD residues 137–254. Each domain consists of a twisted β sandwich, in which the β strands in the opposing β sheets are approximately orthogonal to each other, and a few short α helices. CTD loops (residues 180–188 and residues 208–218) are not visible in the electron density, most probably due to intrinsic disorder in these regions. However, one subunit from each dimer shows no density for residues 170–190, which likely reflects degradation of the protein prior to crystallization, as evidenced by SDS-PAGE (data not shown).

A unique feature of HMPV M among all experimentally solved M proteins so far is the presence of a Ca^2+^ binding site located on the solvent-exposed surface of the NTD. The binding pocket adopts the classic Ca^2+^ pentagonal bipyramidal geometry, with an average coordinating distance of 2.4 Å between Ca^2+^ and interacting oxygens. Binding involves the side chain carboxyls of Glu24 and Asp26, the backbone carbonyl of Leu28 and Lys101, and two water molecules (Figure 1B). The interaction of Asp26 carboxyls is bidentate, and Glu24 shows direct coordination with Ca^2+^ with one of its carboxyl oxygens, whereas the other interacts through a bridging water molecule.

Dimers pack crystallographically through a relatively large (624 Å^2^) NTD/NTD interface (Figure S1A) that is stabilized by polar interactions, propagating a helical arrangement within the crystal. This suggests that this interaction could be involved in the formation of higher order forms of M.

### Structural Characterization of Dimeric HMPV M by SAXS

**Figure 2:**
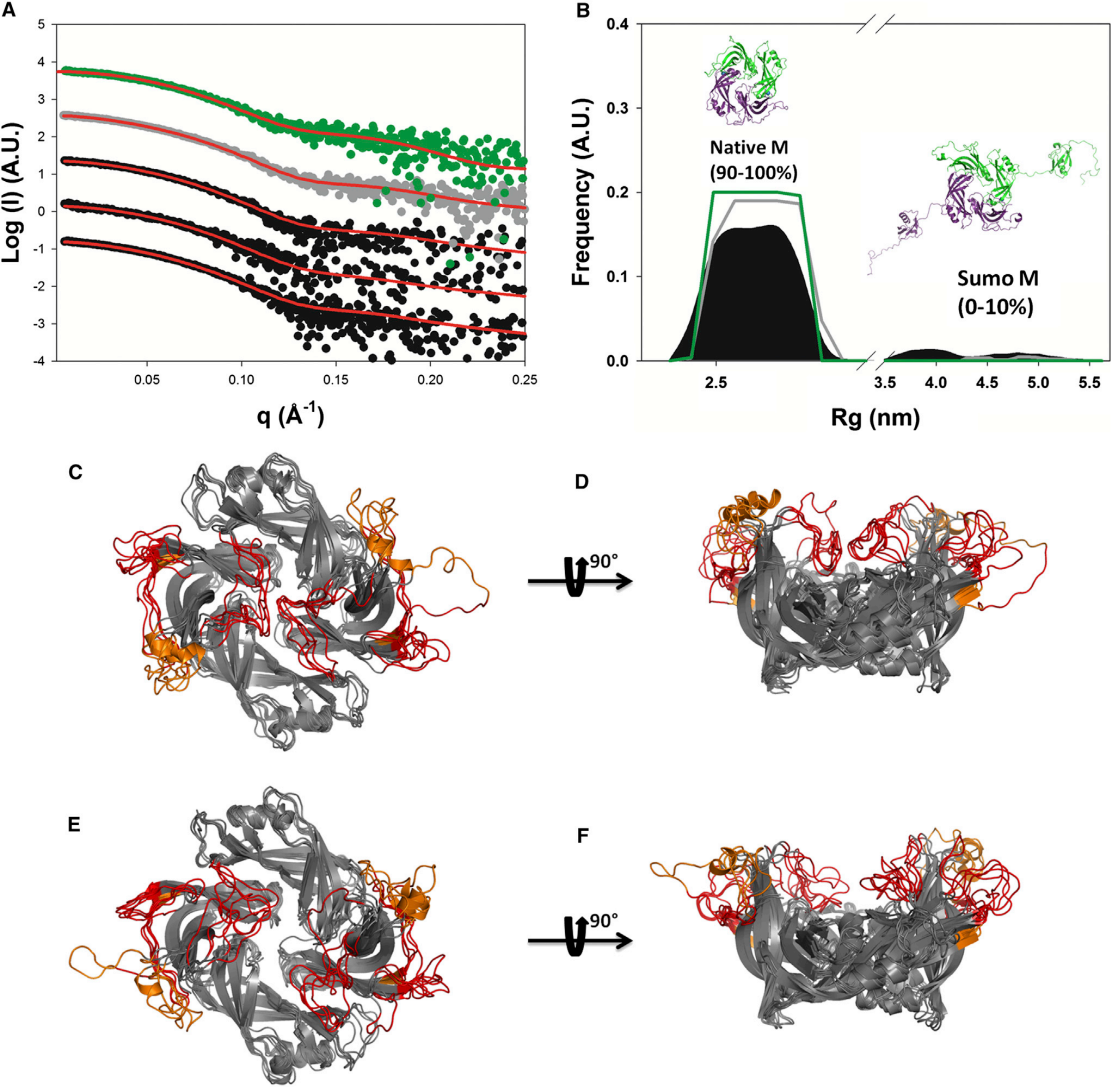
Small-Angle X-Ray Scattering (A) Fitted SAXS profiles of the SUMO-3C-M/untagged M mixture (black spheres) and merged data (gray spheres), measured in 1M non-detergent sulfobetaines 201 (NDSB-201) buffer, and SAXS profile of untagged M in 1M NaCl buffer (green spheres). The red lines represent fits from the OEs. (B) Radius of gyration (R_g_) distributions of the initial pool of 11,000 models (black area), composed of models of untagged M and models bearing one or two N-terminal SUMO-3C tags, and OEs for the SUMO-3C-M/untagged M mixture (gray line) and untagged M (green line). (C and D) Views of five superimposed representative models from the OE corresponding to the SUMO-3C-M/untagged M mixture measured in 1M NDSB-201 buffer. Residues missing from the crystal structure are shown in red, or in orange for residues that are missing in only one monomer. (E and F) Similar views of the OE from untagged M in 1M NaCl. See also Figures S2 and S3.

We investigated the structure of M in solution using SAXS. SUMO-3C-M was measured in its native form and after addition of 1 mM of either Ca^2+^ or EGTA (Figure 2A; see also Figure S2). Oligomeric state and low-resolution structure remained unaffected by the presence of these additives, with Guinier-determined R_g_ values of 4.6–4.8 nm (Figure S2B). Next, we studied untagged M conformation in solution. We first attempted to use untagged M protein purified in 1 M NaCl, which we concentrated directly prior to measurement. However, only a single high-concentration measurement could be obtained (Figure 2A; see also Figure S2), due to protein aggregation occurring within a few hours after concentration. Notably, this aggregation was independent of the addition of 1 mM Ca^2+^ or EGTA (Figure S2C). Because of the intrinsic propensity of HMPV M to both degrade internal loops and self-aggregate at medium to high concentrations, further attempts to produce intact, unaggregated protein used 1 M NDSB-201 containing buffers, resulting in a significant loss of separation power of the S200 column. Thus, M protein could only be obtained as a (9:1 molar ratio) mixture with undigested SUMO-3C-M, as seen on SDS-PAGE (data not shown). Gel filtration fractions were used directly for SAXS measurements and concentrated on site to avoid slow concentration-dependent aggregation of M. Total protein concentrations ranged from 1 to 4 mg/ml, and R_g_ values were between 2.6 and 3.0 nm, as determined by Guinier approximation, depending on the presence or absence of contaminating SUMO-3C-M. These measured R_g_ compared well with the R_g_ value of 2.4 nm calculated from the crystallographic dimer, the 2 Å discrepancy resulting from the absence of loop regions in the crystal. Interestingly, all SAXS profiles showed a marked change in slope at intermediate resolution (∼0.13–0.15Å^−1^), a feature typically observed in hollow macromolecular complexes (as observed in ab initio models built from the SAXS data; Figures S2D and S2E; Ribeiro Ede et al., 2009).

### Ensemble Optimization

The observed SAXS profiles were treated as a mixture of two flexible components (untagged M and SUMO-3C-M). Large ensembles of both proteins were generated by classical MDS (Table S1), which were then fitted to experimental data using EOM in two successive rounds of refinement. The advantage of this approach to analyze flexible mixtures is the combination of MDS ability to fold secondary structure elements missing from the crystal structure with explicit treatment of flexibility in SAXS, resulting in restoration of missing protein fragments and a gain in effective information content (Bernadó et al., 2007, Pelikan et al., 2009). By selecting an optimized ensemble (OE) of MDS models that maximizes the agreement between experimental and simulated SAXS profiles (χ), we obtained a refined model for use in a second round of MDS, using two copies of the most represented monomer as starting coordinates. As an indication of the success of the refinement protocol, the second round of EOM fitting exclusively selected models from the second round of MDS over unrefined models from the first round (data not shown).

Figure 2B shows the R_g_ distribution of pure untagged M and the M/SUMO-3C-M mixture, which forms two well-separated populations. Untagged M represents 90%–100% of the mixture and samples a narrow R_g_ range centered around 2.6 nm, whereas SUMO-M-3C accounts for only 0%–10% of the mixture and samples a wider range of R_g_ (4.0–5.5 nm), consistent with the presence of a flexibly linked SUMO tag. The fit is of good quality and consistent between independent measurements, with χ values comprised between 0.7 and 0.9 (Figure 2A; see also Figure S2). Careful inspection of models in the OEs and comparison with the range of conformations sampled in the pool from MDS indicates that, in solution, M populates only a specific subset of the available MDS-derived conformers. In particular, in presence of 1 M NDSB, the majority of selected models display an interaction between the interdomain linkers, encompassing residues 123–137 (Figures 2C and 2D; see also Figure S3). Untagged M measured in 1 M NaCl buffer tends to show a collapse of the interdomain linkers onto the core of their respective monomers, rather than interaction between them. This difference might be attributable to the stabilizing effect of NDSBs on protein fold (Expert-Bezançon et al., 2003, Vuillard et al., 1998). Furthermore, OEs are enriched in models in which residues 169–174 spontaneously folded into a short α-helical motif during MDS (Figures 2C–2F; see also Figure S3). Notably, a similar α-helix is also present in the same region of RSV M (Money et al., 2009). The OEs display flexibility in the CTD loops connecting β9 and β10 (residues 170–190), and β12 and β13 (residues 208–218), as well as in the interdomain linker (residues 123–137), consistent with the disorder observed in these regions in the crystal structure and in MDS.

### Solution Structure of HMPV M Studied using MDSs

In order to study the impact of bound calcium on the structure, explicit-solvent MDSs were performed in the presence or absence of Ca^2+^ (Table S1) for a total simulation time of ∼2.9 μs. Analysis of atomic root mean square fluctuations (RMSFs) indicated three main flexible regions located in the CTD loops connecting the β sheets (residues 170–190 and 208–218), as well as in the interdomain linker (residues 123–137; Figure S3), in good agreement with the crystal structure. Frequently, folding of a short α-helical motif encompassing residues 169–174 was observed. The interdomain linkers collapsed onto the core of M, consistent with features present in the RSV M crystal structure (Money et al., 2009).

In the crystal structure, Lys106 is involved in stabilizing the relatively large NTD/NTD packing interface through a hydrogen bond with the side chain of Gln77 from a crystallographically related partner (Figure S1C). However, MDS performed in the absence of the bound Ca^2+^ resulted in the formation of an intramolecular salt bridge between Lys106 and Asp26, which otherwise interacts with the Ca^2+^ ion (Figure S1D). This interaction seemed to compensate for the absence of bound Ca^2+^ and suggests that the absence of bound Ca^2+^ might impact negatively on M ultrastructure assembly.

**Figure 3:**
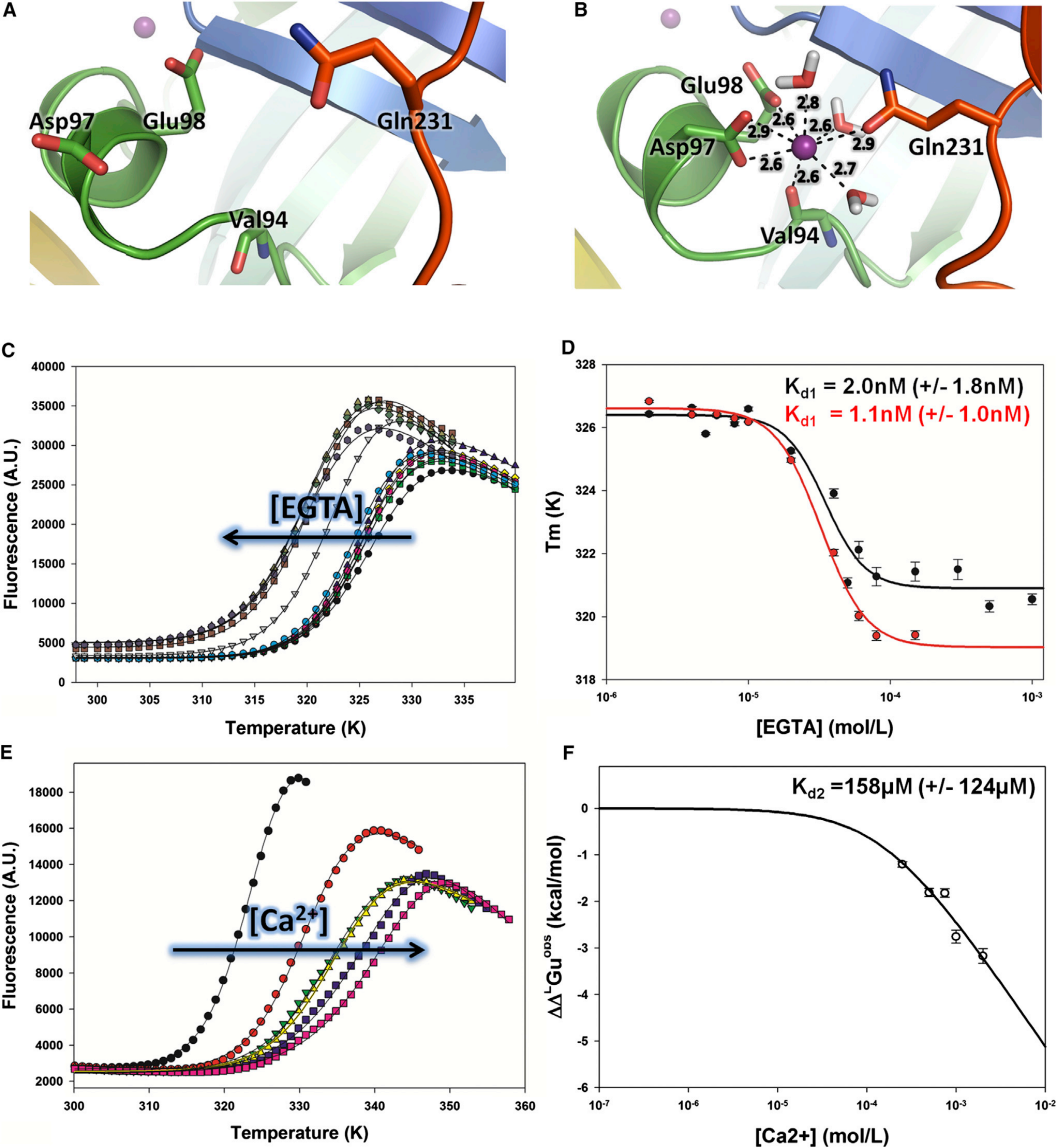
Fluorescence-Based TSA (A) Close-up of the second (low-affinity) Ca^2+^ binding site in the crystal structure. (B) Model of binding of Ca^2+^ as observed in MDS. (C) Unfolding transitions of native SUMO-3C-M (black) in 50 mM HEPES, pH 7.8, and 1.15 M NaCl and with increasing concentrations of EGTA (indicated by an arrow). Only the titration performed using 4 μM of protein is represented for clarity. (D) Plots of *T*_*m*_ versus total EGTA concentration, for a protein concentration of 4 μM (black) or 2 μM (red). The data points were fitted using a four-parameter sigmoidal dose-response function. (E) Unfolding transitions of native SUMO-3C-M (black) in 50 mM HEPES, pH 7.8, and 1.15 M NaCl and with increasing concentrations of CaCl_2_ from 0.25 mM to 2 mM (indicated by an arrow). (F) Plot of ΔΔG_u_ versus total Ca^2+^ concentration. ΔΔG_u_ values were calculated and fitted using equations from Layton and Hellinga (2010). See also Figure S4.

MDSs performed in the presence of 150 mM free calcium in the simulation box predicted binding at a second, lower affinity site, which is more solvent exposed than the primary site and is unoccupied in the crystal structure. Binding of Ca^2+^ in this second site induced slight local conformational changes in α3 and the loop connecting α4 and β13 (Figures 3A and 3B). Additional simulations starting from the bound state in the absence of free Ca^2+^ suggested binding at this low-affinity site was stable (Figure S3C). The geometry of this second Ca^2+^ binding site is shown in Figure 3B, revealing close coordination by Asp97 and Glu98 side chains, the backbone carbonyl of Val94, and the carbonyl group from Gln231 side chain.

### Ca^2+^ Increases M Stability

Thermal shift assays (TSAs) were performed to determine the effect of Ca^2+^ on M stability (Figure S4). Addition of 1 and 5 mM of Ca^2+^ increased the protein melting temperature (*T*_*m*_) from 50.6°C to 68.5°C and 73.8°C, respectively, whereas addition of similar concentrations of EGTA induced a 5°C drop in *T*_*m*_ to 45.5°C. The effect was specific to Ca^2+^, with addition of 5 mM Mg^2+^ leading to no significant change in *T*_*m*_ (Figure S4).

Because SUMO-3C-M is easier to handle and produce in large amounts than untagged M, quantitative TSA binding data were obtained using uncleaved protein. SUMO-3C-M was titrated using either EGTA or CaCl_2_, and unfolding transitions were monitored (Figures 3C and 3E; see also Figure S4), revealing changes in *T*_*m*_ of the same magnitude as observed for untagged M. It is important to emphasize that these variations in *T*_*m*_ did not involve any change in oligomeric state, as evidenced by dynamic light scattering (data not shown) and confirmed by SAXS (Figure S2). Titration of SUMO-3C-M with EGTA could be analyzed by assuming saturation of the crystallographic (high-affinity) Ca^2+^ binding site using the classical Cheng-Prusoff equation (Cheng and Prusoff, 1973) in order to yield the affinity constant of SUMO-3C-M for the first Ca^2+^ binding site (Kd1). Experiments performed at protein concentrations of 2 and 4 μM resulted in values of Kd1 = 1.1 nM (±1.0 nM) or Kd1 = 2.0 nM (±1.8 nM), respectively (Figure 3D; see also Figure S4).

Titration of SUMO-3C-M using Ca^2+^ was analyzed by converting the observed *T*_*m*_ values into Ca^2+^-induced free energy change of unfolding (ΔΔG_u_) following standard procedures (Layton and Hellinga, 2010). Because the SUMO-3C-M used as a reference in this experiment has its high-affinity Ca^2+^ binding site saturated, the analysis can be used to extract the affinity for the second Ca^2+^ binding site (Kd2), yielding a value of Kd2 = 158 μM (±124 μM) (Figure 3F; see also Figure S4). Interestingly, titrations of SUMO-3C-M using EGTA/Ca^2+^ mixtures yielded ΔΔG_u_ versus free Ca^2+^ concentration plots that could not be adequately fitted assuming a single binding site (data not shown), further indicating the presence of the lower affinity site.

### Structure of Lipid-Bound M Filaments

**Figure 4:**
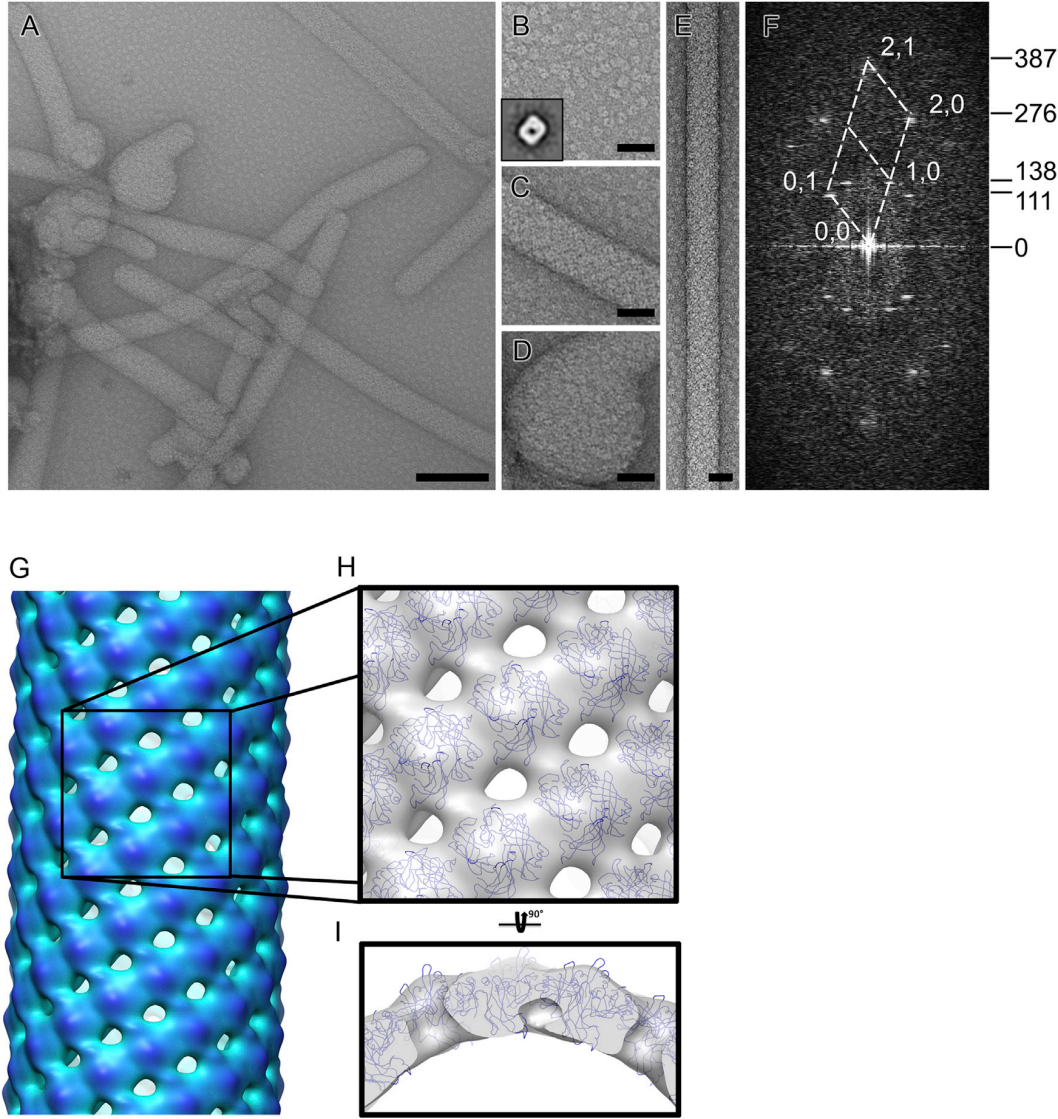
Helical Ordering of M in the Presence of Lipids Visualized by Electron Microscopy (A) Samples of M incubated in the presence of DOPC stained with uranyl acetate revealed tubular and spherical structures with free M in the background. Scale bar = 100 nm. (B) A close-up of free M dimers. Inset shows a class average of M calculated from 577 of 840 particles. (C) A close-up of a tubular filament. M is seen coating the filament surface. (D) A close-up of an M-coated spherical structure. (E) A computationally straightened, long tubule. Scale bars in (B)–(E) = 25 nm. (F) A computational diffraction pattern of the tubule shown in (E) reveals maxima on layer lines, indicating that the tubule has helical symmetry. Lattice indexes (white numbers) and layer line heights (black numbers) are indicated for clearly visible maxima. (G) A radially depth-cued isosurface representation of the density map for lipid-bound M is shown. The map was calculated using helical reconstruction from electron microscopy images of negatively stained samples. (H) A close-up of the map (gray transparent surface) shows the packing of the fitted M (blue) after imposing helical symmetry. Only the C-alpha trace is shown for M. (I) Same rendering as (H), but shown from the side. All isosurfaces were calculated at 2σ above the mean value. See also Figure S5.

Incubation of purified M with 1,2-dioleoyl-sn-glycero-3-phosphocholine (DOPC) resulted, after several days, in the growth of long, flexible tubules with varying diameters (∼37 ± 4 nm), as revealed by electron microscopy (EM; Figure 4). Attempts to study the effect of EGTA and/or calcium on filament formation were inconclusive; the addition of EGTA or calcium led to nonspecific aggregation of the sample over the same time scale required for filament growth.

Images of computationally straightened tubules were analyzed for the presence of higher order organization of M. Fourier transforms of tubule images gave rise to layer lines characteristic of helical symmetry. One tubule (diameter 34 nm) was consistent with principal Bessel orders of 6 and −13 at 1/92 Å and 1/114 Å, respectively (Figure 4F). Helical, three-dimensional reconstruction of this tubule revealed an arrangement of subunits with a rise of 5.16 Å and a turn of −56.6 degrees (Figure 4G).

To model the higher order organization of M, we fitted the X-ray structure, as well as SAXS-validated MD models, into the EM map (Figures 4H and 4I; see also Figure S5) using Chimera (Goddard et al., 2007, Pettersen et al., 2004). The fitting revealed that M can only orientate with its dimeric symmetry axis orthogonal to the long axis of the filament. Most importantly, the fitting unambiguously determined the concave membrane binding surface of the molecule: in all cases, fitting with the concave face of the protein pointing toward lipids resulted in significantly better correlation (C) and overlap (O) between the experimental EM map and a map simulated from the atomic model than fitting with the concave face pointing outside (C = 0.94/O = 126 versus C = 0.88/O = 115; Figure 4I). Furthermore, imposing helical symmetry revealed no clashes between the symmetry-related copies of M providing independent validation for fitting (Figure 4H; see also Figure S4). The packing with the concave face toward the membrane suggests that M polymerization involves side-by-side interactions between lipid-bound subunits (Figure 4H; see also Figure S4), although the resolution of the EM reconstruction was insufficient to precisely define the residues involved in forming these interfaces.

### Structural Comparison with Other ss(−)RNA Viruses Reveals Conservation of Domain Architecture and Interdomain Interfaces across *Paramyxoviridae*

**Figure 5:**
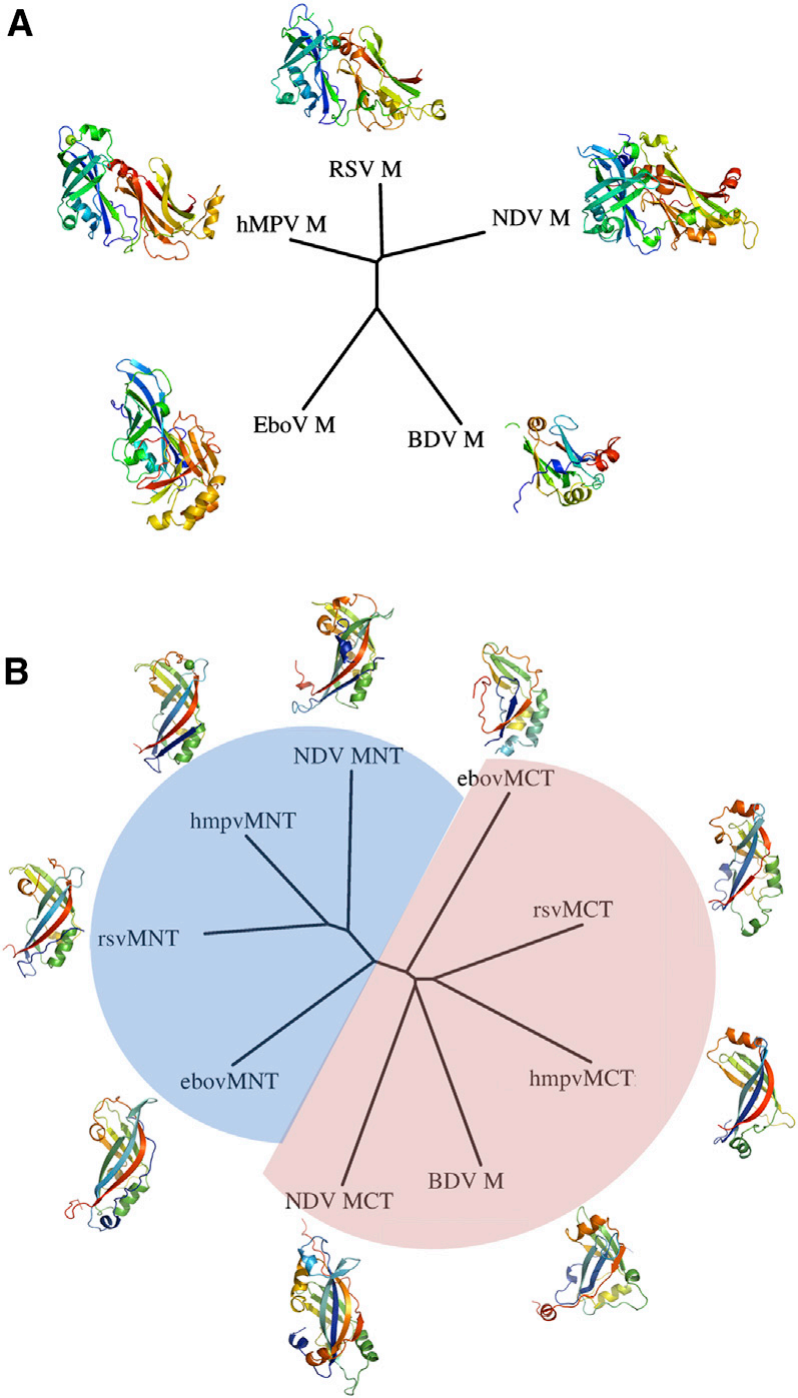
Structural Alignments and Evolutionary Relationships between M Proteins from *Paramyxoviridae*, *Bornaviridae*, and *Filoviridae* (A) Structure-based unrooted phylogenetic tree of known M structures from HMPV, RSV, NDV, EBOV, and BDV. (B) Structure-based unrooted phylogenetic tree of M protein NTD and CTD domains. Evolutionary information was calculated by structural alignment using SHP (Abrescia et al., 2012) and plotting was done using PHYLIP. See also Figure S6.

We used the Structural Homology Program (SHP) to identify evolutionary relationships between M proteins on the basis of structural alignments (Figures 5A and 5B; see also Figure S6). Structural alignments of HMPV, RSV, NDV, and EBOV M indicate that the NTDs and CTDs are homologous across *Paramyxoviridae* and *Filoviridae* families (Figure 5B; see also Figure S6). The location of the NTD/CTD interface is similar in pneumoviruses and avulaviruses, but different in EBOV M, where the CTD adopts a different orientation relative to the NTD (Figures 5A; see also Figure S6). The phylogenetic tree obtained from aligning the five full-length M structures reproduces well the classical distinction observed between *Paramyxoviridae*, *Filoviridae*, and *Bornaviridae*, with a clustering of RSV, HMPV, and NDV M (Figure 5A; see also Figure S6).

Structural comparison at the domain level indicates that BDV M is most similar to the M CTD of *Paramyxoviridae* (Figure 5B), whereas EBOV M NTDs and CTDs cluster with their respective *Paramyxoviridae* counterparts. This partition of NTDs and CTDs implies that the members of *Paramyxoviridae* and *Filoviridae* families evolved from a common ancestor prior to divergence of the structural interdomain relationships in *Filoviridae* M protein. Interestingly, a structure-based sequence alignment of *Filoviridae* and *Pneumovirinae* M proteins reveals a strikingly conserved stretch of residues at the NTD/CTD interface, despite the absence of overall sequence identity. Indeed, the interdomain interaction is mediated in part by a conserved XWXPX motif, where Xs are hydrophobic residues (Figure S6A).

### Similar Quaternary Arrangements Are Observed in Other Paramyxoviruses and in Bornaviruses

**Figure 6:**
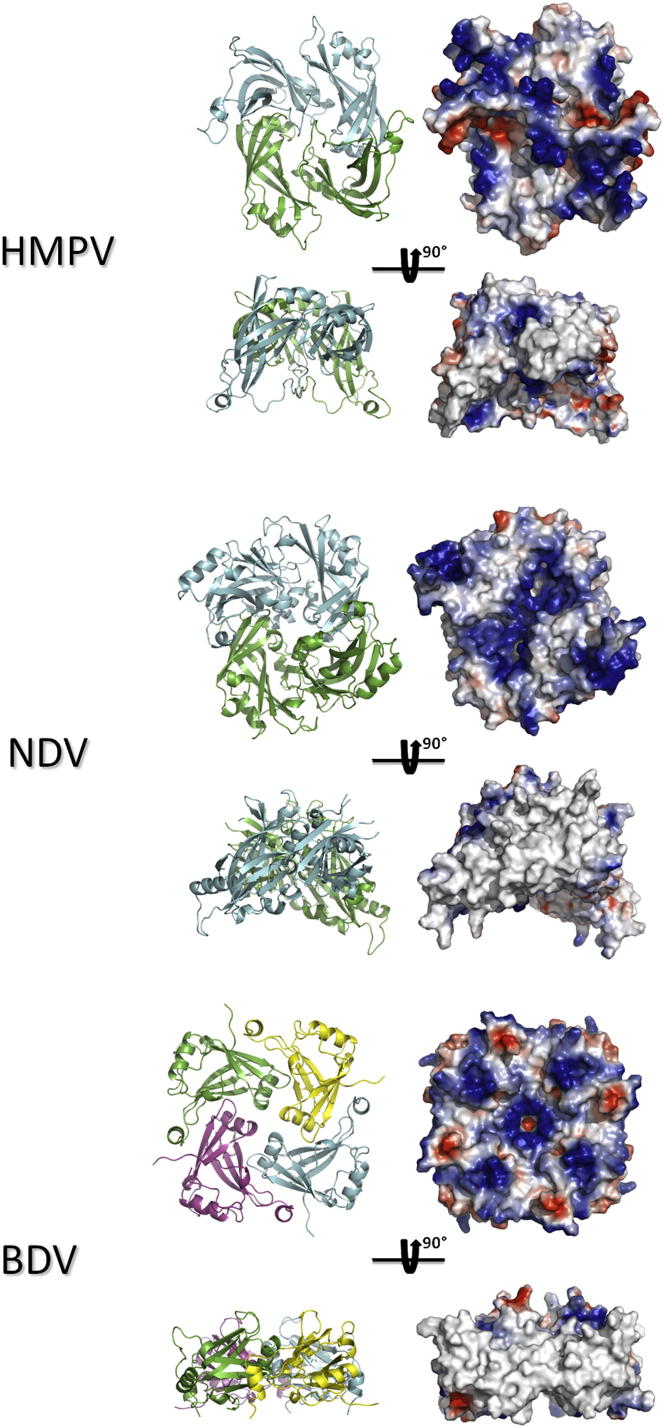
Comparison of the Quaternary Structures of M from HMPV, NDV, and BDV The structures are shown in cartoon and colored by chain. Electrostatic surfaces were drawn using vacuum charges in Pymol.

Figure 6 shows a structural comparison of dimeric HMPV M with dimeric NDV M and tetrameric BDV M. All three M oligomers display a characteristic diamond shape with a central cavity. Electrostatic potential surfaces highlight common features of M proteins, such as the presence of a positively charged face, which is thought to interact with the viral membrane (Money et al., 2009), and a large number of exposed hydrophobic residues on the sides of the diamond-shaped dimer, potentially involved in M-M interactions (Battisti et al., 2012). The similarity in quaternary morphology and surface charges across the M proteins of these viruses suggests that the members of both *Paramyxoviridae* and *Bornaviridae* share a common mode of assembly during viral morphogenesis.

## Discussion

### Metapneumovirus M Forms Dimers in the Crystal and in Solution

The data presented here demonstrates that HMPV M forms dimers, both in the crystalline state and in solution, as shown by our MDS-based ensemble optimization approach to fitting of the SAXS data (Figure 2; see also Figure S2). In addition, the diamond-shaped particles observed in EM (Figure 4B) are reminiscent of the crystal structure (Figure 1; see also Figure S1). The monomeric subunits of M are formed by two consecutive β sandwich domains, connected by a flexible linker. The dimer is stabilized by a large conserved hydrophobic interface, giving rise to a diamond-shaped molecule, with a concave and a convex surface. The concave surface exposes positively charged flexible loops that are thought to interact with the viral membrane (Money et al., 2009). Together with the observed structural similarity to the previously reported dimeric NDV M (Battisti et al., 2012) but also for tetrameric BDV M (Neumann et al., 2009), this indicates that the M dimers are the basic unit for matrix assembly in these viruses.

### Implications of the Structure of M Filaments for Viral Assembly and Budding

The helical filament of DOPC-bound HMPV M provides experimental evidence that M interacts with lipids via its concave face. Interestingly, the electrostatic surface of the concave face of M comprises negatively charged residues that are partially covered by positively charged loops (Figure 6), resulting in a surface that is complementary to the zwitterionic choline heads that harbor a terminal quaternary ammonium followed by a phosphate group. Notably, helical assemblies with a diameter of 29 nm were reported for RSV M following prolonged incubation with 1,2-dipalmitoyl-sn-glycero-3-phosphoethanolamine/DOPC mixtures (McPhee et al., 2011; versus a diameter of 37 ± 4 nm for HMPV M filaments), highlighting the similarity in the lipid binding and self-assembly properties between the two proteins.

Additionally, the uncharged sides of the protein are involved in formation of contacts between M dimers, predominantly through NTD/NTD interfaces. We note that the positive curvature observed in the filament structure is likely to be nonphysiological since the M dimers coat the exterior of the lipid tubules. The same nonphysiological curvature has been observed with RSV (Liljeroos et al., 2013, McPhee et al., 2011). Interestingly, the filaments revealed similar side-to-side contacts as in the crystal structure of M (Figure S1A). However, the packing of M in the crystal displays the opposite curvature to that seen in the EM filaments (Figure S5). This negative curvature perhaps recapitulates more closely the packing and assembly of M in the virion. The observation that M can form filaments with different curvatures suggests that this dynamic plasticity in M packing might play a role in HMPV morphogenesis. In RSV virions, the presence of an inner layer of membrane-associated M protein has been shown to correlate with partial ordering of the glycoprotein spikes (Liljeroos et al., 2013), suggesting that in the context of viral infection, M would bind membranes at sites enriched in viral glycoproteins and the concave face would be involved in binding the conserved cytoplasmic tails of F and/or G proteins. This indicates that the formation of flexible, curved, or planar arrays of M proteins directly controls the localization and impacts the conformation of F and G proteins within the membrane, allowing membrane deformations required for budding to take place without disruption of viral particles. Thus, we could postulate that the plasticity of M protein self-assemblies enables M to transduce internal signals from the cell cytoplasm, such as conformational change induced by nucleocapsid binding, leading to a change in M array curvature, membrane deformation, and budding.

### Effect of Ca^2+^ Binding on M Stability

A distinguishing feature of metapneumovirus, and perhaps pneumovirus M proteins, resides in their ability to bind Ca^2+^, as evidenced by the presence of Ca^2+^ in the X-ray structure, changes in *T*_*m*_ observed by TSA, and observations from MDS. Many viruses have been shown to perturb Ca^2+^ homeostasis and utilize Ca^2+^ and cellular Ca^2+^-binding proteins in their replication cycles (reviewed in Zhou et al., 2009). In particular, paramyxoviruses such as Sendai virus have been reported to increase cytosolic Ca^2+^ concentrations, leading to a rounding of chicken erythrocytes and increased rates of cell fusion (Hallett et al., 1982, Volsky and Loyter, 1978). RSV replication in cell culture was also negatively impacted by the absence of Ca^2+^, and syncytium formation was inhibited (Shahrabadi and Lee, 1988). Interestingly, the SH protein of RSV, a viroporin specific to the *Pneumovirinae*, associates with cellular membranes and forms pentameric, cation-selective channels in infected cells (Carter et al., 2010, Gan et al., 2008, Gan et al., 2012), possibly leading to increased cytosolic Ca^2+^ levels.

The residues involved in side chain coordination to Ca^2+^ for both the high- and low-affinity sites are conserved between RSV and HMPV (Figure S6B), suggesting a common utilization of Ca^2+^ in these viruses. However, the binding sites seem to have diverged in other pneumoviruses, such as pneumonia virus of mice and pneumonia virus of dog (data not shown). Intriguingly, the high-affinity Ca^2+^ binding pocket in RSV crystal structures (PDB ID 2VQP and 2YKD) is in an open conformation, and the α3 helix that forms the binding site is unresolved, raising the possibility of cleavage or conformational disorder, possibly induced by the use of chelating agents.

Because the Ca^2+^ binding sites are located on the convex face of M, it is possible that the variations in Ca^2+^ concentrations inside infected cells at various stages of the viral cycle regulate the assembly of viral nucleocapsids onto M arrays at viral budding sites, but also perhaps intracellular transport of M proteins to the membrane. Additionally, exposure of viral particles to low calcium concentrations after cell entry could play a role in the uncoating of nucleocapsids from the inner matrix layer of the virion. Finally, the observed 25°C difference in the thermal stability of unbound and Ca^2+^-bound HMPV M at 1 mM Ca^2+^ suggests that Ca^2+^ is involved in stabilizing virions in the Ca^2+^-rich extracellular environment, thus improving virion lifetime and infectivity.

### Evolution of *Mononegavirales* M Proteins

Analysis of structural relationships among M proteins from members of *Paramyxoviridae*, *Bornaviridae*, and *Filoviridae* reveals structural similarity of the NTDs and CTDs across these families and provides direct evidence that EBOV, NDV, and HMPV/RSV evolved from a common ancestor prior to divergence of the families and changes to the quaternary structure of M. Gene duplication took place prior to separation of these viruses, suggesting that BDV, which possesses a single-domain M protein, might be more similar to the common ancestor of these three *Mononegavirales* families. Additionally, the clustering of BDV M with CTDs of the other M proteins implies that the CTD was the originator and that the NTD appeared later through duplication. This observation is consistent with the fact that membrane binding of EBOV matrix protein VP40 (EBOV M) occurs primarily through its CTD (Ruigrok et al., 2000). Indeed, EBOV M assembles into ring-like structures that bind lipid membranes (Ruigrok et al., 2000, Scianimanico et al., 2000, Timmins et al., 2003) and induces budding of virus-like particles when expressed in the absence of any other viral protein (Timmins et al., 2001). Interestingly, the structural relationship between EBOV M NTDs and CTDs in the crystal structure is different to that observed in the HMPV, RSV, and NDV matrix proteins (Figure 5A; Figure S9). It has been suggested that the NTDs and CTDs from EBOV M can move relative to each other and that M is in equilibrium between alternative oligomeric states, such as monomers (Dessen et al., 2000, Scianimanico et al., 2000), dimers (Timmins et al., 2003), hexamers (Ruigrok et al., 2000, Scianimanico et al., 2000, Timmins et al., 2003), and octamers (Gomis-Rüth et al., 2003). Specifically, the formation of an octameric RNA binding ring of NTDs has been associated with a separate function of M during the viral cycle, further supporting the hypothesis of divergence of NTD function after gene duplication from a CTD ancestor. Recently structural studies of EBOV VP40 have revealed a number of different oligomerization interfaces (Bornholdt et al., 2013). In HMPV M, the dimeric building block is formed by NTD/CTD interactions between monomers that form the dimer and occludes as surface area that is twice as large as any interface observed in EBOV VP40. In both cases, NTD/NTD and CTD/CTD interfaces are the basis for higher order assemblies. However, the main difference resides in the nature of the minimal building block, which in EBOV is a monomer and in HMPV is a dimer (Bornholdt et al., 2013).

Intriguingly, RNA binding capability has also been reported for RSV M (Rodríguez et al., 2004), raising the possibility that HMPV M might interact with RNA as well. However, several of the residues involved in RNA binding in RSV are not conserved in HMPV M (Figure S2), indicating that this function might not be shared between different members of the *Pneumovirinae*.

### Conclusions

We have shown that HMPV M forms Ca^2+^-binding dimers with a concave and a convex face. The dimers assemble onto lipids via their concave face and form various higher order structures through side-by-side interactions. Calcium appears to be critical for M stability, and is potentially involved in regulating processes such as viral entry, uncoating, assembly, and budding. This suggests that the Ca^2+^-binding pockets are potential targets for the development of small-molecule inhibitors. HMPV M shares a common shape and similar surface charge distributions with the other members of the *Paramyxoviridae* and *Bornaviridae*, suggesting a common mode of self-assembly. Taken together, these results further our understanding of metapneumovirus morphogenesis and evolution.

## Experimental Procedures

### Protein Cloning, Expression, and Purification

The HMPV M gene from strain NL1-00 was cloned into pOPINS3C (Berrow et al., 2007) for expression of M with an N-terminal SUMO-3C cleavage site using a proprietary ligation-independent In-Fusion System (Clontech) following standard procedures. The integrity of the cloned construct was checked by nucleotide sequencing.

The SUMO-3C-M construct was expressed in Rosetta2 *E. coli* cells by overnight incubation under shaking at 17°C following 1 mM IPTG induction of 1 l terrific broth in presence of appropriate antibiotics. Cells were harvested by centrifugation (18°C, 20 min, 4000 x *g*). Cell pellets were resuspended in 20 mM Tris, pH 7.5, 1 M NaCl and lyzed by sonication. The lysate was then centrifuged for 45 min at 4°C and 50000 x *g*. The supernatant was filtered and loaded on a column containing pre-equilibrated Ni-NTA Agarose (QIAGEN). After extensive washes, the protein was eluted in 20 mM Tris, pH 7.5, 1 M NaCl, 400 mM imidazole. Size exclusion chromatography was then run on an S200 column equilibrated in 20 mM Tris, pH 7.5, 1 M NaCl. The SUMO tag was removed by addition of 3C protease at 4°C for 72 h. The cleaved product was further purified through reverse Ni-NTA purification to remove Histagged 3C protease followed by an additional gel filtration step (either in 20 mM Tris, pH 7.5, 1 M NaCl, or in 20 mM Tris, pH 7.5, 5 mM dithiothreitol, 650 mM NaCl, 1M NDSB-201). The protein was concentrated using a Millipore concentration unit (cut off 10 kDa) in presence of 1 M NDSB-201 in order to avoid M protein aggregation and/or precipitation at concentrations above ∼1 mg/ml.

### Crystallization and Data Collection

Crystallization was carried out via the vapor diffusion method using a Cartesian Technologies pipetting system (Walter et al., 2005). The M protein crystallized after ∼28 days in 20% polyethylene glycol 6000, 100 mM Tris, pH 8.0, 10 mM zinc chloride at 20°C. Crystals were frozen in liquid nitrogen after being soaked in a mother liquor solution supplemented with 25% glycerol. Diffraction data were recorded on the I03 beamline at Diamond Light Source. All data were automatically processed by xia2 (Winter et al., 2013).

### Structure Determination and Refinement

Structural determination was initiated by molecular replacement using RSV M (PDB ID 2VQP) as a search model in PHASER (McCoy et al., 2007). The solution was subjected to rounds of restrained refinement in PHENIX (Adams et al., 2010) and Autobuster (Blanc et al., 2004) and manual building in COOT (Emsley et al., 2010). TLS parameters were included in the final round of refinement. The CCP4 program suite (Winn et al., 2011) was used for coordinate manipulations. The structures were validated with Molprobity (Chen et al., 2010). Refinement statistics are given in Table 1, and final refined coordinates and structure factors have been deposited in the PDB with accession code 4LP7.

### Structure Analysis

All the structure-related figures were prepared with the PyMOL Molecular Graphics System (DeLano Scientific). Electrostatic potential calculations were performed with APBS tools (Baker et al., 2001). Protein interfaces were analyzed with the PISA webserver (Krissinel and Henrick, 2007). Structure-based sequence alignments were performed using PROMALS 3D (Pei et al., 2008). Structural alignments were calculated using SHP (Stuart et al., 1979).

### Small-Angle X-Ray Scattering Experiments

Small-angle X-ray scattering measurements for cleaved M and M/SUMO-3C-M mixtures were performed at the BM29 beamline in the European Synchrotron Radiation Facility (ESRF). Data were collected at 20°C, a wavelength of 0.0995 nm, and a sample-to-detector distance of 1 m. The 1D scattering profiles were generated, and blank subtraction was performed by the data processing pipeline available at BM29 at the ESRF. Additional data for SUMO-3C-M were collected at the ID22 beamline at Diamond Light Source. The scattering profile of untagged M was analyzed using GNOM (Svergun, 1992) to yield the pair distribution function P(r) (Figure S2), twenty independent ab initio reconstructions were generated using DAMMIF (Franke and Svergun, 2009), and the models were averaged using DAMAVER (Volkov and Svergun, 2003).

### MD and Ensemble Optimization

Starting coordinates for the missing residues of M and SUMO-3C-M were added in extended conformations in Modeler (Eswar et al., 2008). Coordinates for the SUMO tag were taken from PDB entry 3UF8. All MD simulations were performed using GROMACS 4 (Hess et al., 2008) and the AMBER99SB-ILDN^∗^ force field (Best and Hummer, 2009, Lindorff-Larsen et al., 2010). At the beginning of each simulation, the protein was immersed in a box of extended simple point charge water, with a minimum distance of 1.0 nm between protein atoms and the edges of the box. A total of 150 mM NaCl was added using genion. Long-range electrostatics were treated with the particle-mesh Ewald summation (Essmann et al., 1995). Bond lengths were constrained using the P-LINCS algorithm. The integration time step was 5 femtoseconds. The v-rescale thermostat and the Parrinello-Rahman barostat were used to maintain a temperature of 300 K and a pressure of 1 atm. Each system was energy minimized using 1,000 steps of steepest descent and equilibrated for 200 ps with restrained protein heavy atoms. For each system, two independent production simulations were obtained by using different initial velocities. The aggregated simulation time was ∼2.9 μs for M and ∼0.4 μs for SUMO-3C-M. RMSFs were calculated using GROMACS routines. Snapshots were extracted every 100 ps, resulting in a pool of ∼12,000 models. Theoretical SAXS patterns were calculated with the program CRYSOL (Svergun et al., 1995), and ensemble fitting was performed with GAJOE (Bernadó et al., 2007).

### Thermal Shift Assay

The TSA (ThermoFluor) was carried out in a real-time PCR machine (BioRad DNA Engine Opticon 2), where buffered solutions of protein and fluorophore (SYPRO Orange; Molecular Probes; Invitrogen), with and without additives, were heated in a stepwise fashion from 20°C to 99°C at a rate of 1°C/min. An appropriate volume of protein and 3 μl of SYPRO Orange (Molecular Probes; Invitrogen) were made up to a total assay volume of 50 μl with starting buffer (50 mM HEPES, pH 7.8, 1.1 M NaCl) in white, low-profile, thin-wall PCR plates (Abgene) sealed with microseal “B” films (BioRad). The fluorophore was excited in the range of 470–505 nm and fluorescence emission was measured in the range of 540–700 nm every 0.5°C after a 10 s hold. The effect of Ca^2+^ ions was assayed by comparing *T*_*m*_ of protein in starting buffer and in EGTA or CaCl_2_ supplemented buffer. All thermal shift reactions were performed in triplicate. *T*_*m*_, enthalpy of unfolding (ΔH_u_), and change in heat capacity upon Ca^2+^ binding were calculated by fitting the experimental data to Equations 9, 24, and 25 from Layton and Hellinga (2010). Effective concentration values were extracted from EGTA titration curves by fitting the data to a four-parameter sigmoidal dose-response curve, and Kd1 was calculated by using the Cheng-Prusoff equation (Cheng and Prusoff, 1973), assuming a value of 17.2 nM for Kd(EGTA-Ca^2+^) at the *T*_*m*_, in presence of 1.1 M NaCl at pH 7.8 (calculated using the MAXCHELATOR server http://maxchelator.stanford.edu/).

### Electron Microscopy and Image Processing

Purified HMPV M (7.2 μM) was incubated with DOPC (400 μM; Avanti Polar Lipids) for 7 days in +37°C. Electron microscopy grids of the mixture were stained with 2% uranyl acetate. Images were taken on CCD (UltraScan 4000SP, Gatan) with a transmission electron microscope (Tecnai F30, FEI) operated at 200 kV and at 39,000× nominal magnification, resulting in a calibrated pixel size of 3.1 Å/pixel. Contrast transfer function estimation and phase flipping were carried out using XMIPP (http://xmipp.cnb.csic.es/), and the rest of the analysis using Burnham-Brandeis Helical Package (http://coan.burnham.org/other-projects/brandeis-helical-package/). Extracted and straightened filaments were Fourier transformed for assigning layer-line heights and Bessel orders followed by three-dimensional reconstruction (Owen et al., 1996). The map was solvent-flattened in the lipid and solvent parts. Atomic models of M were fitted into the electron microscopy map in UCSF Chimera (Pettersen et al., 2004), and helical symmetry was applied on the fitted structure using Bsoft (Heymann et al., 2008). The electron microscopy reconstruction has been deposited in the Electron Microscopy Data Bank (EMD-2415). Two-dimensional class averages of unbound M were calculated in Relion (Scheres, 2012).

## Author Contributions

C.L. and J.M.G. conceived and designed research. C.L., M.R., and J.T.H. performed experiments. C.L., J.T.H., and J.M.G. analyzed data. C.L., J.T.H., and J.M.G. wrote the paper.
