# Supplementary material for: Structure and Self-Assembly of the Calcium Binding Matrix Protein of Human Metapneumovirus
Source: Structure. 2014 Jan 7;22(1):136–48. doi: 10.1016/j.str.2013.10.013 (PMC3887258; doi:10.1016/j.str.2013.10.013)
Supplement: Document S1. Figures S1–S6 and Table S1 [file mmc1.pdf]

Structure, Volume *22*

Supplemental Information

Structure and Self-Assembly of the Calcium Binding Matrix Protein of Human Metapneumovirus

Cedric Leyrat, Max Renner, Karl Harlos, Juha T. Huiskonen, and Jonathan M. Grimes

Supplementary Information

Table S1, related to Figure 2 and 3: summary of MD simulations.

System	$[Ca^{2+}]_{free}$ (mol/L)	System size (atoms)	Total simulation time (µs)
Unrefined M + crystallographic Ca^{2+}	0	79,920	0.51
Refined M + crystallographic Ca^{2+}	0	71,503	0.64
Refined M – no Ca^{2+}	0	71,509	0.55
Refined M + crystallographic Ca^{2+}	0.15	71,367	0.51
Refined M + crystallographic Ca^{2+}+ low affinity Ca^{2+}	0	71,488	0.64

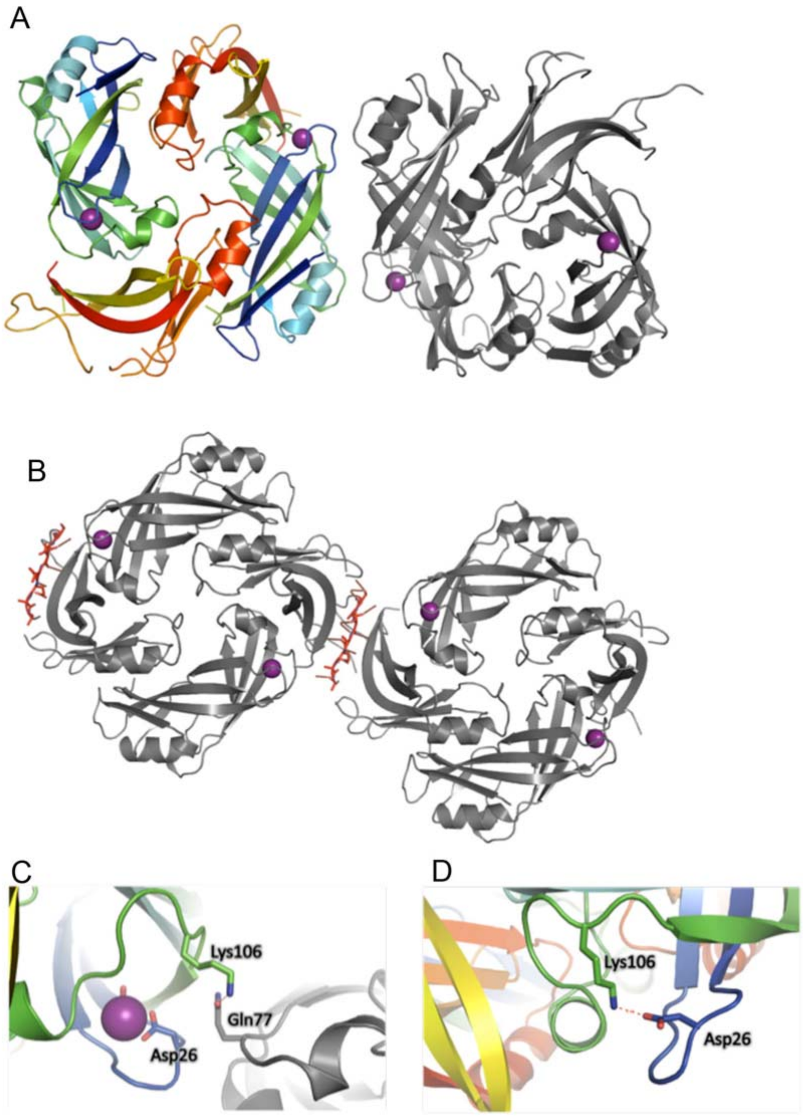

Supplementary figure 1, related to Figure 1: Structure of the asymmetric unit, crystal contacts and interaction of Lys106 with Gln77 and Asp26. A. Cartoon representation of the two dimers that form the crystal asymmetric unit, showing the 624 Å^2 NTD/NTD interface. One monomer is coloured from blue (N-terminus) to red (C-terminus), while the other is in gray. Ca^{2+} ions are shown as purple spheres. B. Crystal contacts involving M CTDs, showing how the strand formed by residues 174–180 (in orange) clips onto the adjacent strand from a crystallographically related molecule. C. Close up of the polar contact between the side chains of Lys106 and Gln77 from a crystallographically related molecule in the crystal structure. D. Salt bridge formed between Lys106 and Gln77 during MDS in the absence of bound Ca^{2+}.

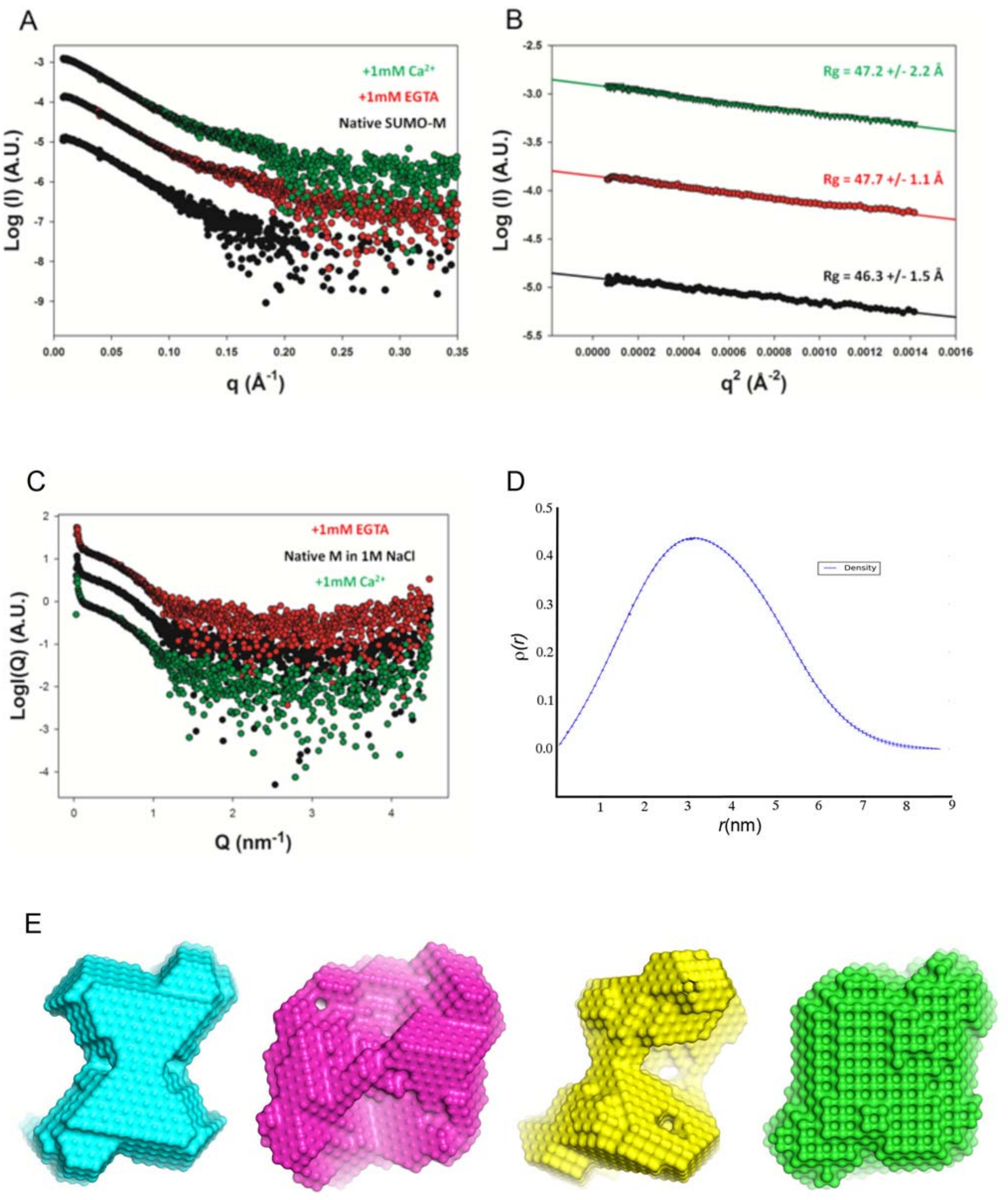

Supplementary figure 2, related to Figure 2: Solution studies by SAXS. A. SAXS profiles showing the effect of Ca^{2+} and EGTA on SUMO-3C-M solution structure studied by SAXS. B. Corresponding Guinier plots. C SAXS profiles of untagged M. The profiles show aggregation of concentrated untagged M in 1 M NaCl buffer, occurring within a few hours after concentration of the sample. D Pair distribution function P(r) calculated from the SAXS profile of untagged M in 1M NaCl buffer (Figure 1A, green spheres). E Dammif *ab-initio* models of untagged M calculated from D, imposing 2-fold symmetry. Three representative models are shown (coloured cyan, purple and yellow) along with the averaged model (in green) of 20 independent reconstructions.

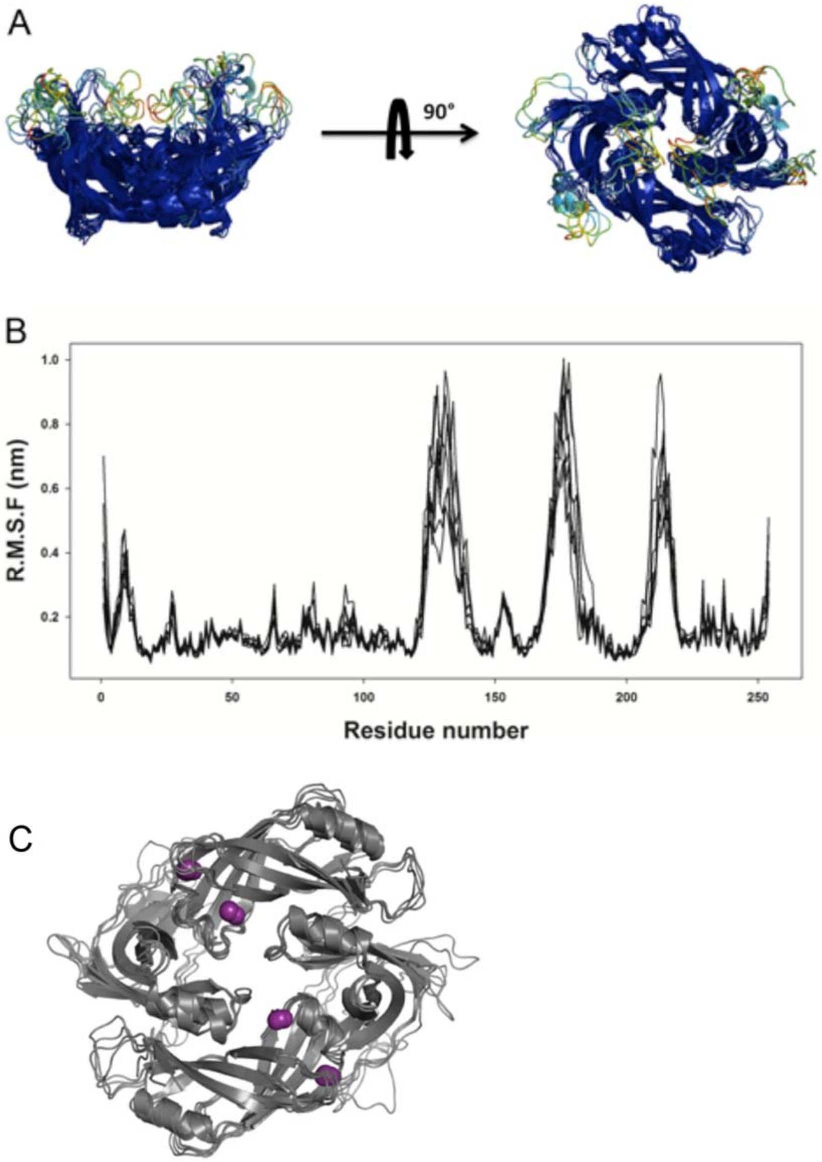

Supplementary figure 3, related to Figure 2: Root mean square fluctuations (RMSF) extracted from MDS. Residue-averaged RMSF were calculated using refined M trajectories (see Table S1) after structural alignment in GROMACS. The first 50 ns were excluded from the calculation. A. Mapping of RMSF onto superimposed final snapshots from refined M simulations (system 2 to 5 from Table S1). B. RMSF versus Residue number plot. C Stability of the bound Calcium ions by MDS. Superimposed snapshots from MDS taken at 60 ns interval, demonstrating the stability of Ca^{2+} binding at the second site in the absence of free Ca^{2+} in the simulation box (System 5 from Table S1). The protein is shown in gray cartoons and the Ca^{2+} ions are displayed as purple spheres.

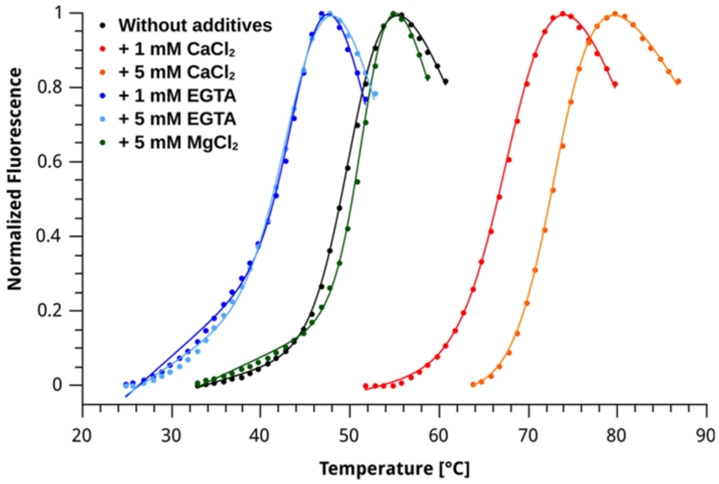

	Tm [°C]	Δ Tm [°C]
**Without additives**	50.57 ± 0.34	-
**+ 1 mM $CaCl_2$**	68.49 ± 0.43	+ 17.93
**+ 5 mM $CaCl_2$**	73.81 ± 0.81	+ 23.25
**+ 1 mM EGTA**	45.51 ± 0.60	− 5.06
**+ 5 mM EGTA**	45.24 ± 1.25	− 5.33
**+ 5 mM $MgCl_2$**	51.23 ± 0.64	+ 0.66

Supplementary figure 4, related to Figure 3: Unfolding transitions of untagged M in its native state, or in presence of EGTA, $CaCl_2$ or $MgCl_2$. T_m values shown were calculated by applying a Boltzmann distribution equation about the sigmoidal melting curves to obtain the inflection point (the midpoint of the unfolding transition) of the slope (GraphPad Prism software). Values in parentheses indicate the standard error for calculated T_m values. An increase in T_m indicates a stabilization of the protein by an increase in structural order and a reduction in conformational flexibility, while a decrease in T_m indicates a destabilization.

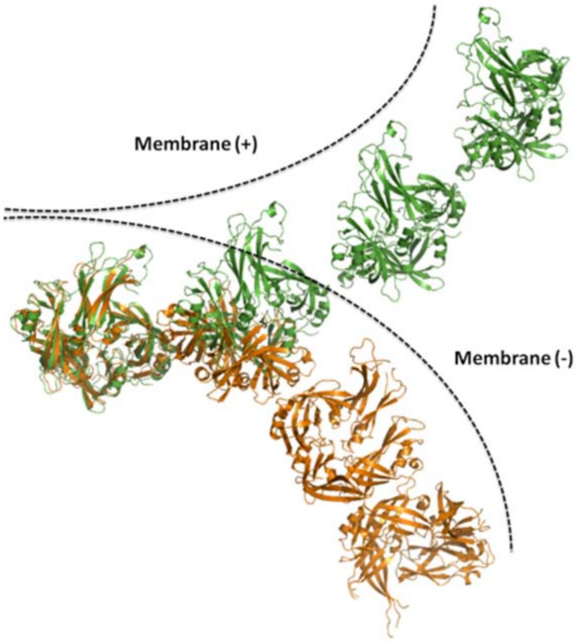

Supplementary figure 5, related to Figure 4: Conformational plasticity of M–M interactions. Comparison of helical arrangements observed in the crystal (orange) and in the lipid-bound M filament (green), illustrating the opposite curvatures (indicated by (+) and (-)). The membrane surface is represented by a black dotted line.

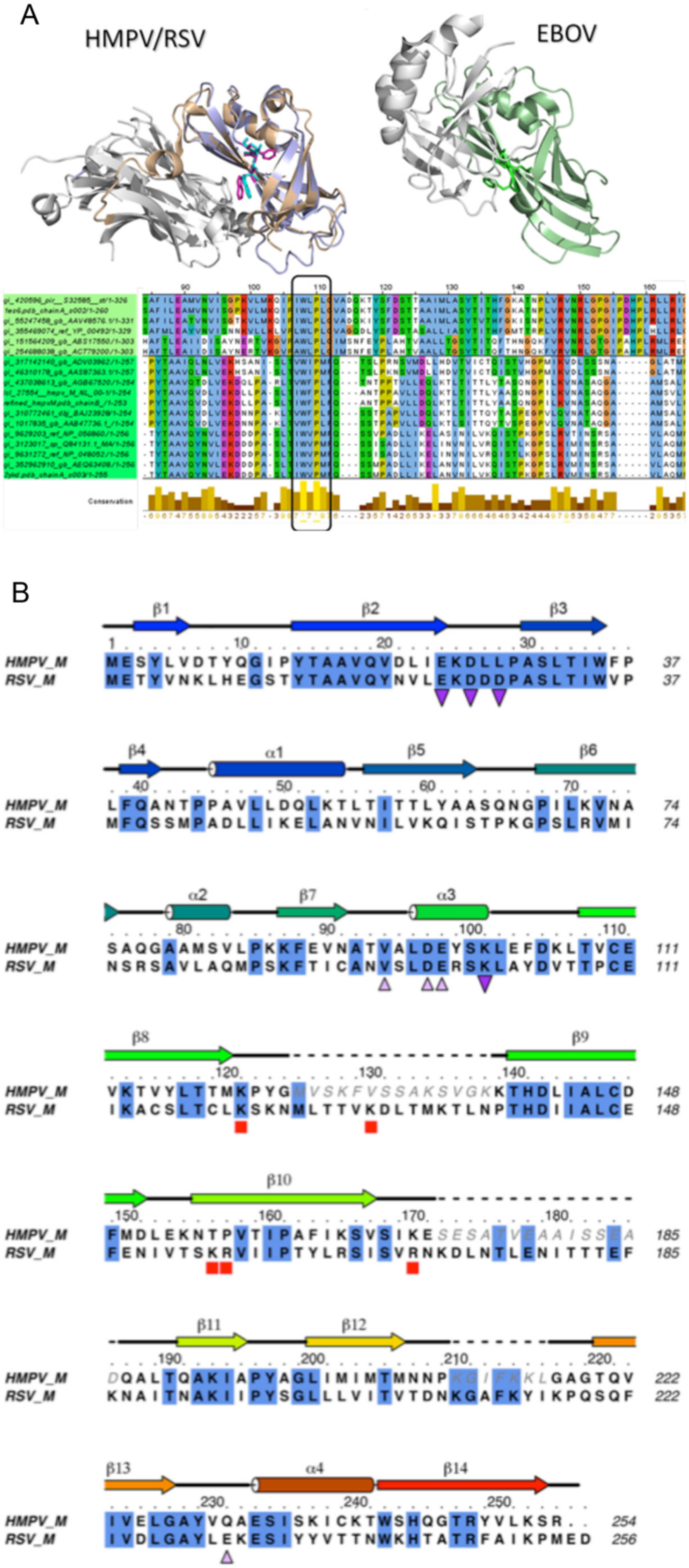

Supplementary figure 6, related to Figure 5: Sequence alignments. A. PROMALS3D alignment of *Pneumovirinae* and *Filoviridae* M sequences using the known structures of HMPV, RSV and EBOV. The structure superimpositions and sequence alignment highlight the conservation of an hydrophobic xWxPx motif located in the NTD across the viral families and which is involved in interdomain contacts. M CTD is shown in white cartoons. HMPV and RSV M NTDs are represented in purple and wheat cartoons, respectively. EBOV MNTD is shown in green. The WxP motif is shown as sticks.

B Structure based sequence alignment of HMPV and RSV M proteins. Annotated secondary structure is drawn from blue to red, the residues forming the high affinity Ca^{2+} binding site are marked by inverted purple triangles, and the low affinity site marked in light purple. The RNA binding residues of RSV M are marked by red squares.
